# Correction: A Higher Level Classification of All Living Organisms

**DOI:** 10.1371/journal.pone.0130114

**Published:** 2015-06-11

**Authors:** Michael A. Ruggiero, Dennis P. Gordon, Thomas M. Orrell, Nicolas Bailly, Thierry Bourgoin, Richard C. Brusca, Thomas Cavalier-Smith, Michael D. Guiry, Paul M. Kirk

There are a number of errors in Tables 1 and 2. Please see the corrected Tables 1 and 2 here. The S1 Table Supporting Information file incorrectly spells "Pseudanabaenales” and is also corrected here.

**Table 1:** List of ranks used in the hierarchy with the number of taxa per rank

Rank	Number of Taxa
Superkingdom	2
**Kingdom**	**7**
Subkingdom	11
Infrakingdom	8
Superphylum	6
**Phylum**	**96**
Subphylum	60
Infraphylum	4
Superclass	12
**Class**	**352**
Subclass	145
Infraclass	23
Superorder	52
**Order**	**1,468**

Main ranks are in bold type; unnamed taxa are not counted.

**Table 2:** Proposed hierarchical classification from superkingdom to order.

**SUPERKINGDOM PROKARYOTA**													
	**KINGDOM ARCHAEA [= ARCHAEBACTERIA]**												
					Phylum Crenarchaeota								
									Class “Aigarchaeota”				
													Order N.N. ("*Ca*. *Caldiarchaeum"*)
									Class “Korarchaeota”				
													Order N.N. (*"Ca*. *Korarchaeum"*)
									Class “Thaumarchaeota”				
													Order Cenarchaeales
									Class Thermoprotei [= Crenarchaeota]				
													Order Acidilobales
													Order Desulfurococcales
													Order Fervidicoccales
													Order Sulfolobales
													Order Thermoproteales
					Phylum Euryarchaeota								
									Class Archaeoglobi				
													Order Archaeoglobales
									Class Halobacteria				
													Order Halobacteriales
									Class Methanobacteria				
													Order Methanobacteriales
									Class Methanococci				
													Order Methanococcales
									Class “Methanomicrobia”				
													Order Methanocellales
													Order Methanomicrobiales
													Order Methanosarcinales
									Class Methanopyri				
													Order Methanopyrales
									Class “Nanohaloarchaea”				
													Order N.N. (e.g., *"Ca*. *Nanosalinarum"*)
									Class Thermococci				
													Order Thermococcales
									Class Thermoplasmata				
													Order Thermoplasmatales
	**KINGDOM BACTERIA [= EUBACTERIA]**												
		SUBKINGDOM NEGIBACTERIA											
					Phylum Acidobacteria								
									Class N.N. (*Bryobacter*)				
									Class Acidobacteria				
													Order Acidobacteriales
									Class Holophagae				
													Order Acanthopleuribacterales
													Order Holophagales
					Phylum Aquificae								
									Class Aquificae				
													Order Aquificales
					Phylum Armatimonadetes								
									Class Armatimonadia				
													Order Armatimonadales
									Class Chthonomonadetes				
													Order Chthonomonadales
									Class Fimbriimonadia				
													Order Fimbriimonadales
					Phylum Bacteroidetes								
									Class Bacteroidia				
													Order Bacteroidales
									Class Cytophagia				
													Order Cytophagales
									Class Flavobacteria				
													Order Flavobacteriales
									Class Sphingobacteriia				
													Order Sphingobacteriales
					Phylum Caldiserica								
									Class Caldisericia				
													Order Caldisericales
					Phylum Chlamydiae								
									Class Chlamydiae				
													Order Chlamydiales
					Phylum Chlorobi								
									Class Chlorobea				
													Order Chlorobiales
									Class Ignavibacteria				
													Order Ignavibacteriales
					Phylum Chrysiogenetes								
									Class Chrysiogenetes				
													Order Chrysiogenales
					Phylum Cyanobacteria [= Cyanophyta]								
									Class Cyanophyceae [= Phycobacteria]				
													Order Chroococcales
													Order Nostocales
													Order Oscillatoriales
													Order Pseudanabaenales
													Order Synechococcales
									Class Gloeobacteria [= Gloeobacterophyceae]				
													Order Gloeobacterales
					Phylum Deferribacteres								
									Class Deferribacteres				
													Order Deferribacterales
					Phylum Deinococcus-Thermus [= Hadobacteria]								
									Class Deinococci				
													Order Deinococcales
													Order Thermales
					Phylum Dictyoglomi								
									Class Dictyoglomia				
													Order Dictyoglomales
					Phylum Elusimicrobia								
									Class Elusimicrobia				
													Order Elusimicrobiales
					Phylum Fibrobacteres								
									Class Fibrobacteria				
													Order Fibrobacterales
					Phylum Fusobacteria								
									Class Fusobacteriia				
													Order Fusobacteriales
					Phylum Gemmatimonadetes								
									Class Gemmatimonadetes				
													Order Gemmatimonadales
					Phylum Lentisphaerae								
									Class Lentisphaeria				
													Order Lentisphaerales
													Order Victivallales
									Class Oligosphaeria				
													Order Oligosphaerales
					Phylum Nitrospira								
									Class "Nitrospira"				
													Order "Nitrospirales"
					Phylum Planctomycetes								
									Class Phycisphaerae				
													Order Phycisphaerales
									Class Planctomycea				
													Order Planctomycetales
					Phylum Proteobacteria								
									Class Alphaproteobacteria				
													Order N.N. (e.g., *Breoghania*)
													Order Caulobacterales
													Order Kiloniellales
													Order Kordiimonadales
													Order Magnetococcales
													Order “Parvularculales”
													Order Rhizobiales
													Order Rhodobacterales
													Order Rhodospirillales
													Order Rickettsiales
													Order Sneathiellales
													Order Sphingomonadales
									Class Betaproteobacteria				
													Order N.N. (*Chitinivorax*)
													Order Burkholderiales
													Order Hydrogenophilales
													Order Methylophilales
													Order Neisseriales
													Order Nitrosomonadales
													Order "Procabacteriales"
													Order Rhodocyclales
									Class Deltaproteobacteria				
													Order N.N. (e.g., *Deferrisoma*)
													Order Bdellovibrionales
													Order Desulfarculales
													Order Desulfobacterales
													Order Desulfovibrionales
													Order Desulfurellales
													Order Desulfuromonadales
													Order Myxococcales
													Order Syntrophobacterales
									Class Epsilonproteobacteria				
													Order Campylobacterales
													Order Nautiliales
									Class Gammaproteobacteria				
													Order N.N. (e.g., *Alkalimonas*)
													Order Acidithiobacillales
													Order Aeromonadales
													Order Alteromonadales
													Order Cardiobacteriales
													Order Chromatiales
													Order “Enterobacteriales”
													Order Legionellales
													Order Methylococcales
													Order Oceanospirillales
													Order Orbales
													Order Pasteurellales
													Order Pseudomonadales
													Order “Salinisphaerales”
													Order Thiotrichales
													Order “Vibrionales”
													Order Xanthomonadales
									Class Zetaproteobacteria				
													Order Mariprofundales
					Phylum Spirochaetae								
									Class "Spirochaetes"				
													Order Spirochaetales
					Phylum Synergistetes								
									Class Synergistia				
													Order Synergistales
					Phylum Thermodesulfobacteria								
									Class Thermodesulfobacteria				
													Order Thermodesulfobacteriales
					Phylum Thermotogae								
									Class Thermotogae				
													Order Thermotogales
					Phylum Verrucomicrobia								
									Class Opitutae				
													Order Opitutales
													Order Puniceicoccales
									Class Verrucomicrobiae				
													Order Verrucomicrobiales
		SUBKINGDOM POSIBACTERIA											
					Phylum Actinobacteria								
									Class Actinobacteria				
													Order Acidimicrobiales
													Order Actinomycetales
													Order Bifidobacteriales
													Order Coriobacteriales
													Order Euzebyales
													Order Gaiellales
													Order Nitriliruptorales
													Order Rubrobacterales
													Order Solirubrobacterales
													Order Thermoleophilales
					Phylum Chloroflexi [= Chlorobacteria]								
									Class Anaerolineae				
													Order Anaerolineales
									Class Caldilineae				
													Order Caldilineales
									Class Chloroflexia				
													Order Chloroflexales
													Order Herpetosiphonales
									Class Dehalococcoidia				
													Order Dehalococcoidales
									Class Ktedonobacteria				
													Order Ktedonobacterales
													Order Thermogemmatisporales
									Class Thermomicrobia				
													Order Sphaerobacterales
													Order Thermomicrobiales
					Phylum Firmicutes								
									Class Bacilli				
													Order Bacillales
													Order Lactobacillales
									Class Clostridia				
													Order Clostridiales
													Order Halanaerobiales
													Order Natranaerobiales
													Order Thermoanaerobacterales
									Class Erysipelotrichia				
													Order Erysipelotrichales
									Class Negativicutes				
													Order Selenomonadales
									Class Thermolithobacteria				
													Order Thermolithobacterales
					Phylum Tenericutes								
									Class Mollicutes				
													Order Acholeplasmatales
													Order Anaeroplasmatales
													Order Entomoplasmatales
													Order Haloplasmatales
													Order Mycoplasmatales
**SUPERKINGDOM EUKARYOTA**													
	**KINGDOM PROTOZOA**												
		SUBKINGDOM EOZOA											
			INFRAKINGDOM EUGLENOZOA										
					Phylum Euglenozoa								
						Subphylum N.N.							
									Class Diplonemea				
													Order Diplonemida
									Class Kinetoplastea				
													Order Bodonida
													Order Prokinetoplastida
													Order Trypanosomatida
						Subphylum Euglenoida							
									Class N.N.				
													Order Petalomonadida
													Order Ploeotiida
									Class Euglenophyceae				
													Order Euglenida
													Order Eutreptiida
									Class Peranemea				
													Order Heteronemida
													Order Peranemida
													Order Rhabdomonadida
						Subphylum Symbiontida							
									Class Postgaardea				
													Order Postgaardida
			INFRAKINGDOM EXCAVATA										
					Phylum Loukozoa								
						Subphylum Eolouka							
									Class Jakobea				
													Order Jakobida
									Class Tsukubea				
													Order Tsukubamonadida
						Subphylum Neolouka							
									Class Malawimonadea				
													Order Malawimonadida
					Phylum Metamonada								
									Class Anaeromonadea				
													Order Oxymonadida
													Order Trimastigida
									Class Carpomonadea				
													Order Carpediemonadida
													Order Chilomastigida
													Order Dysnectida
									Class Eopharyngea				
													Order Diplomonadida
													Order Retortamonadida
									Class Trichomonadea				
													Order Cristamonadida
													Order Spirotrichonymphida
													Order Trichomonadida
													Order Tritrichomonadida
									Class Trichonymphea				
													Order Lophomonadida
													Order Trichonymphida
					Phylum Percolozoa								
						Subphylum Pharyngomonada							
									Class Pharyngomonadea				
													Order Pharyngomonadida
						Subphylum Tetramitia							
									Class Heterolobosea				
													Order Acrasida
													Order Schizopyrenida
									Class Lyromonadea				
													Order Lyromonadida
									Class Percolatea				
													Order Percolomonadida
													Order Pseudociliatida
		SUBKINGDOM SARCOMASTIGOTA											
					Phylum Amoebozoa								
						Subphylum Conosa							
									Class Archamoebea				
													Order Mastigamoebida
													Order Pelobiontida
													Order Rhizomastigida
									Class Dictyostelea				
													Order Dictyostelida
									Class Myxogastrea [= Myxomycetes]				
										Subclass Exosporeae			
													Order Ceratiomyxida
										Subclass Myxogastria			
												Superorder Collumelidia	
													Order Echinosteliida
													Order Fuscisporida
												Superorder Lucisporidia	
													Order Liceida
													Order Trichiida
									Class Protostelea				
													Order Protostelida
									Class Variosea				
													Order Artodiscida
													Order Holomastigida
													Order Phalansteriida
													Order Varipodida
						Subphylum Lobosa							
									Class Discosea				
										Subclass Flabellinia			
													Order Dactylopodida
													Order Himatismenida
													Order Pellitida
													Order Stygamoebida
													Order Trichosida
													Order Vanellida
										Subclass Longamoebia			
													Order Dermamoebida
													Order Centramoebida
													Order Thecamoebida
									Class Tubulinea [= Lobosea]				
													Order Arcellinida
													Order Echinamoebida
													Order Euamoebida
													Order Leptomyxida
													Order Nolandida
					Phylum Choanozoa [with Microsporidia, Animalia, and Fungi constitutes "Supergroup Opisthokonta"]								
						Subphylum Choanofila							
									Class Choanoflagellatea				
													Order Acanthoecida
													Order Craspedida
									Class Corallochytrea				
													Order Corallochytrida
									Class Filasterea				
													Order Ministeriida
									Class Ichthyosporea				
													Order Dermocystida
													Order Eccrinida
						Subphylum Paramycia							
									Class Aphelidea				
													Order Aphelidida
									Class Cristidiscoidea				
													Order Fonticulida
													Order Nucleariida
									Class Rozellidea				
													Order Rozellida
					Phylum Microsporidia [with Choanozoa, Animalia, and Fungi constitutes "Supergroup Opisthokonta"]								
									Class Disporea				
													Order N.N. (e.g., *Nosema*)
									Class Metchnikovellea				
													Order Metchnikovellida
									Class Minisporea [= Microsporea]				
													Order Minisporida [= Minisporea]
									Class Pleistophorea				
													Order Pleistophorida
					Phylum Sulcozoa								
						Subphylum Apusozoa							
									Class Breviatea				
													Order Breviatida
									Class Thecomonadea				
													Order Apusomonadida
						Subphylum Varisulca							
									Class Diphyllatea				
													Order Diphylleida
									Class Glissodiscea				
													Order Mantamonadida
													Order Planomonadida
									Class Hilomonadea				
													Order Rigifilida
	**KINGDOM CHROMISTA**												
		SUBKINGDOM HACROBIA											
					Phylum N.N.								
									Class Endohelea				
													Order Heliomonadida
													Order Microhelida
									Class Picomonadea				
													Order Picomonadida
									Class Telonemea				
													Order Telonemida
					Phylum Cryptista								
						Subphylum Palpitia							
									Class Palpitea				
													Order Palpitida
						Subphylum Rollomonadia							
									Class Cryptophyceae				
													Order Cryptomonadales
													Order Pyrenomonadales
													Order Tetragonidiales
									Class Goniomonadea				
													Order Goniomonadida
									Class Leucocryptea				
													Order Kathablepharida
					Phylum Haptophyta								
									Class Coccolithophyceae [= Prymnesiophyceae]				
													Order Coccolithales
													Order Coccosphaerales
													Order Isochrysidales
													Order Phaeocystales
													Order Prymnesiales
													Order Syracosphaerales
													Order Zygodiscales
									Class Pavlovophyceae				
													Order Pavlovales
					Phylum Heliozoa								
									Class Centrohelea				
													Order Acanthocystida
													Order Pterocystida
		SUBKINGDOM HAROSA [= "Supergroup SAR"]											
			INFRAKINGDOM HALVARIA										
				Superphylum Alveolata									
					Phylum Ciliophora								
						Subphylum Intramacronucleata							
									Class Armophorea				
													Order Armophorida
													Order Clevelandellida
									Class Colpodea				
													Order Bryometopida
													Order Bryophryida
													Order Bursariomorphida
													Order Colpodida
													Order Cyrtolophosidida
													Order Sorogenida
									Class Litostomatea				
										Subclass Haptoria			
													Order Cyclotrichiida
													Order Haptorida
													Order Pleurostomatida
										Subclass Trichostomatia			
													Order Entodiniomorphida
													Order Macropodiniida
													Order Vestibuliferida
									Class Spirotrichea				
										Subclass Choreotrichia			
													Order Tintinnida
										Subclass Hypotrichia			
													Order Euplotida
													Order Kiitrichida
										Subclass Licnophoria			
													Order Licnophorida
										Subclass Oligotrichia			
													Order Strombidiida
										Subclass Protocruziidia			
													Order Phacodiniida
													Order Protocruziida
										Subclass Stichotrichia			
													Order Sporadotrichida
													Order Stichotrichida
													Order Urostylida
									Class Nassophorea				
													Order Colpodidiida
													Order Microthoracida
													Order Nassulida
													Order Synhymeniida
									Class Oligohymenophorea				
										Subclass Apostomatia			
													Order Apostomatida
													Order Astomatophorida
													Order Pilisuctorida
										Subclass Astomatia			
													Order Astomatida
										Subclass Hymenostomatia			
													Order Ophyroglenida
													Order Tetrahymenida
										Subclass Peniculia			
													Order Peniculida
													Order Urocentrida
										Subclass Peritrichia			
													Order Mobilida
													Order Sessilida
										Subclass Scuticociliatia			
													Order Philasterida
													Order Pleuronematida
													Order Thigmotrichida
									Class Phyllopharyngea				
										Subclass Chonotrichia			
													Order Cryptogemmida
													Order Exogemmiida
										Subclass Cyrtophoria			
													Order Chlamydodontida
													Order Dysteriida
										Subclass Rhynchodia			
													Order Hypocomatida
													Order Rhynchodida
										Subclass Suctoria			
													Order Endogenida
													Order Evaginogenida
													Order Exogenida
									Class Plagiopylea				
													Order Odontostomatida
													Order Plagiopylida
									Class Prostomatea				
													Order Prorodontida
													Order Prostomatida
						Subphylum Postciliodesmatophora							
									Class Heterotrichea				
													Order Heterotrichida
									Class Karyorelictea				
													Order Loxodida
													Order Protoheterotrichida
													Order Protostomatida
					Phylum Miozoa								
						Subphylum Myzozoa							
							Infraphylum Apicomplexa						
								Superclass Apicomonada					
									Class Apicomonadea				
													Order Chromerida
													Order Colpodellida
													Order Voromonadida
								Superclass Sporozoa					
									Class N.N.				
													Order Blastogregarinida
									Class Coccidiomorphea				
										Subclass Coccidia			
													Order Adeleida
													Order Eimeriida
													Order Ixorheida
										Subclass Coelotrophia			
													Order Coelotrophiida [= Protococcidiida]
										Subclass Hematozoa			
												Superorder Aconoidia	
													Order Nephromycida
													Order Piroplasmida
												Superorder Haemosporidia	
													Order Haemosporida
									Class Gregarinomorphea				
										Subclass Cryptogregaria			
													Order Cryptogregarida (*Cryptosporidium*)
										Subclass Histogregaria			
													Order Histogregarida
										Subclass Orthogregarinia			
													Order Arthrogregarida
													Order Vermigregarida
									Class Paragregarea				
													Order Archigregarinida
													Order Stenophorida
													Order Velocida
							Infraphylum Dinozoa						
								Superclass Dinoflagellata					
									Class Dinophyceae				
										Subclass N.N.			
													Order Actiniscales
													Order Blastodinales
													Order Coccidinales
													Order Dinamoebales
													Order Lophodinales
													Order Pyrocystales
													Order Thoracosphaerales
										Subclass Dinophysoidia			
													Order Dinophysidales
													Order Nannoceratopsales
										Subclass Gonyaulacoidia			
													Order Gonyaulacales
													Order Gymnodiniales
										Subclass Peridinoidia			
													Order Peridiniales
													Order Prorocentrales
										Subclass Suessioidia			
													Order Suessiales
									Class Ellobiopsea				
													Order Ellobiopsida
									Class Noctilucea				
													Order Noctilucida
									Class Oxyrrhea				
													Order Acrocoelida
													Order Oxyrrhida
									Class Syndinea				
													Order Rastrimonadida
													Order Syndinida
								Superclass Perkinsozoa					
									Class Myzomonadea				
													Order Algovorida
									Class Perkinsea				
													Order Perkinsida
													Order Phagodinida
									Class Squirmidea				
													Order Squirmida
						Subphylum Protalveolata							
									Class Colponemea				
													Order Colponemida
				Superphylum Heterokonta [= "Supergroup Stramenopiles"]									
					Phylum Bigyra								
									Class Bikosea				
													Order Anoecida
													Order Bicoecida
													Order Borokida
													Order Pseudodendromonadida
													Order Rictida
									Class Blastocystea				
													Order Blastocystida
									Class Nanomonadea				
													Order Uniciliatida
									Class Opalinea				
													Order Opalinida
													Order Proteromonadida
									Class Labyrinthulea				
													Order Labyrinthulida
													Order Thraustochytriida
									Class Placididea [= Placidiophyceae]				
													Order Placidiida
					Phylum Ochrophyta [= Heterokontophyta p.p.]								
									Class Bacillariophyceae [= Diatomeae]				
										Subclass Bacillariophycidae			
													Order Achnanthales
													Order Bacillariales
													Order Cymbellales
													Order Dictyoneidales
													Order Eunotiales
													Order Lyrellales
													Order Mastogloiales
													Order Naviculales
													Order Rhopalodiales
													Order Surirellales
													Order Thalassiophysales
										Subclass Coscinodiscophycidae			
													Order Anaulales
													Order Arachnoidiscales
													Order Asterolamprales
													Order Aulacoseirales
													Order Biddulphiales
													Order Chaetocerotales
													Order Chrysanthemodiscales
													Order Corethrales
													Order Coscinodiscales
													Order Cymatosirales
													Order Ethmodiscales
													Order Hemiaulales
													Order Leptocylindrales
													Order Lithodesmiales
													Order Melosirales
													Order Orthoseirales
													Order Paraliales
													Order Rhizosoleniales
													Order Stictocyclales
													Order Stictodiscales
													Order Thalassiosirales
													Order Triceratiales
										Subclass Fragilariophycidae			
													Order Ardissoneales
													Order Climacospheniales
													Order Cyclophorales
													Order Fragilariales
													Order Licmophorales
													Order Protoraphidales
													Order Rhabdonematales
													Order Rhaphoneidales
													Order Striatellales
													Order Tabellariales
													Order Thalassionematales
													Order Toxariales
									Class Bolidophyceae				
													Order Parmales [= Bolidomonadales]
									Class Chrysomerophyceae				
													Order Chrysomeridales
									Class Chrysophyceae				
													Order Chloramoebales
													Order Chromulinales
													Order Chrysosphaerales
													Order Heterogloeales
													Order Hibberdiales
													Order Hydrurales
													Order Ochromonadales
													Order Paraphysomonadida
													Order Synurales
													Order Thallochrysidales
									Class Eustigmatophyceae				
													Order Eustigmatales
									Class Dictyochophyceae [= Hypogyristea]				
													Order Dictyochales
													Order Olisthodiscales
													Order Pedinellales
													Order Pelagomonadales
													Order Sarcinochrysidales
									Class Phaeophyceae				
										Subclass Dictypophycidae			
													Order Dictyotales
													Order Onslowiales
													Order Sphacelariales
													Order Syringodermatales
										Subclass Discosporangiophycidae			
													Order Discosporangiales
										Subclass Fucophycidae			
													Order Ascoseirales
													Order Asterocladales
													Order Desmarestiales
													Order Ectocarpales
													Order Fucales
													Order Laminariales
													Order Nemodermatales
													Order Phaeosiphoniellales
													Order Ralfsiales
													Order Scytothamnales
													Order Sporochnales
													Order Tilopteridales [= Cutleriales]
										Subclass Ishigeophycidae			
													Order Ishigeales
									Class Phaeothamniophyceae [= Aurophyceae]				
													Order Aurearenales
													Order Phaeothamniales
									Class Picophagophyceae [= Picophagea]				
													Order Picophagales
													Order Synchromales
									Class Pinguiophyceae				
													Order Pinguiochrysidales
									Class Raphidophyceae				
													Order Actinophryida
													Order Commatiida
													Order Raphidomonadales
									Class Schizocladiophyceae				
													Order Schizocladiales
									Class Xanthophyceae				
													Order Mischococcales
													Order Pleurochloridellales
													Order Tribonematales
													Order Vaucheriales
					Phylum Pseudofungi [= Oomycota]								
									Class Bigyromonadea				
													Order Developayellida
									Class Hyphochytrea				
													Order Hyphochytriida
													Order Pirsoniida
									Class Oomycetes				
										Subclass Eogamia			
													Order Anisolpidiales
													Order Haptoglossales
													Order Lagenismatales
													Order Olpidiopsidales
													Order Rozellopsidales
										Subclass Peronosporidae			
													Order Peronosporales
													Order Pythiales
													Order Rhipidiales
										Subclass Saprolegniidae			
													Order Albuginales
													Order Leptomitales
													Order Salilagenidiales
													Order Saprolegniales
			INFRAKINGDOM RHIZARIA										
					Phylum Cercozoa								
						Subphylum Endomyxa							
									Class Ascetosporea				
													Order Claustrosporida
													Order Haplosporida
													Order Paradinida
													Order Paramyxida
									Class Gromiidea				
													Order Gromiida
													Order Reticulosida
									Class Phytomyxea				
													Order Phagomyxida
													Order Plasmodiophorida
									Class Vampyrellidea				
													Order Vampyrellida
						Subphylum Monadofilosa							
									Class Imbricatea				
										Subclass Placonuda			
													Order Discocelida
													Order Discomonadida
													Order Euglyphida
													Order Marimonadida
													Order Variglissida
										Subclass Placoperla			
													Order Perlofilida
													Order Rotosphaerida
													Order Spongomonadida
													Order Thaumatomonadida
													Order Zoelucasida
									Class Metromonadea				
													Order Metopiida
													Order Metromonadida
									Class Sarcomonadea				
													Order Cercomonadida
													Order Glissomonadida
													Order Pansomonadida
													Order Pseudosporida
													Order Sainouroida
									Class Thecofilosea				
										Subclass Eothecia			
													Order Cryomonadida
													Order Ebriida
													Order Matazida
													Order Ventricleftida
										Subclass Phaeodaria			
													Order Eodarida
													Order Opaloconchida
										Subclass Tectosia			
													Order Tectofilosida
						Subphylum Reticulofilosa							
									Class Chlorarachnea				
													Order Chlorarachnida
									Class Granofilosea				
													Order Cryptofilida
													Order Desmothoracida
													Order Leucodictyida
													Order Limnofilida
									Class Skiomonadea				
													Order Tremulida
					Phylum Retaria								
						Subphylum Foraminifera							
									Class Monothalamea				
													Order Allogromiida
													Order Astrorhizida
													Order Psamminida
													Order Stannomida
									Class Globothalamea				
													Order Carterinida
													Order Globeriginida
													Order Lagenida
													Order Lituolida
													Order Lofusiida
													Order Robertinida
													Order Rotaliida
													Order Testulariida
													Order Trochamminida
									Class Tubothalamea				
													Order Miliolida
													Order Spirillinida
						Subphylum Radiozoa							
								Superclass Polycystinia					
									Class Polycystinea				
													Order Collodarida
													Order Nassellaria
													Order Spumellaria
								Superclass Spasmaria					
									Class Acantharea				
													Order Arthracanthida
													Order Chaunacanthida
													Order Holacanthida
													Order Symphyacanthida
									Class Sticholonchea				
													Order Taxopodida
	**KINGDOM FUNGI**												
		SUBKINGDOM DIKARYA [= NEOMYCOTA]											
					Phylum Ascomycota								
						Subphylum Pezizomycotina							
									Class Archaeorhizomycetes				
													Order Archaeorhizomycetales
													Order Lahmiales
													Order Triblidiales
									Class Arthoniomycetes				
													Order Arthoniales
									Class Dothideomycetes				
										Subclass N.N.			
													Order Acrospermales
													Order Botryosphaeriales
													Order Hysteriales
													Order Jahnulales
													Order Koralionastetales
													Order Patellariales
													Order Trypetheliales
										Subclass Dothideomycetidae			
													Order Capnodiales
													Order Dothideales
													Order Microthyriales
													Order Myriangiales
										Subclass Meliolomycetidae			
													Order Meliolales
										Subclass Pleosporomycetidae			
													Order Mytilinidales
													Order Pleosporales
									Class Eurotiomycetes				
										Subclass Chaetothyriomycetidae			
													Order Chaetothyriales
													Order Pyrenulales
													Order Verrucariales
										Subclass Eurotiomycetidae			
													Order Arachnomycetales
													Order Ascosphaerales
													Order Coryneliales
													Order Eurotiales
													Order Onygenales
										Subclass Mycocaliciomycetidae			
													Order Mycocaliciales
									Class Laboulbeniomycetes				
													Order Laboulbeniales
													Order Pyxidiophorales
									Class Lecanoromycetes				
										Subclass N.N.			
													Order Candelariales
													Order Umbilicariales
										Subclass Acarosporomycetidae			
													Order Acarosporales
										Subclass Lecanoromycetidae			
													Order Lecanorales
													Order Lecideales
													Order Peltigerales
													Order Rhizocarpales
													Order Teloschistales
										Subclass Ostropomycetidae			
													Order Agyriales
													Order Baeomycetales
													Order Ostropales
													Order Pertusariales
									Class Leotiomycetes				
													Order Cyttariales
													Order Erysiphales
													Order Geoglossales
													Order Helotiales
													Order Leotiales
													Order Mediolariales
													Order Rhytismatales
													Order Thelebolales
									Class Lichinomycetes				
													Order Eremithallales
													Order Lichinales
									Class Orbiliomycetes				
													Order Orbiliales
									Class Pezizomycetes				
													Order Pezizales
									Class Sordariomycetes				
										Subclass N.N.			
													Order Phyllachorales
													Order Trichosphaeriales
										Subclass Hypocreomycetidae			
													Order Coronophorales
													Order Hypocreales
													Order Melanosporales
													Order Microascales
										Subclass Sordariomycetidae			
													Order Boliniales
													Order Calosphaeriales
													Order Chaetosphaeriales
													Order Coniochaetales
													Order Diaporthales
													Order Ophiostomatales
													Order Sordariales
										Subclass Spathulosporomycetidae			
													Order Lulworthiales
										Subclass Xylariomycetidae			
													Order Xylariales
						Subphylum Saccharomycotina							
									Class Saccharomycetes				
													Order Saccharomycetales
						Subphylum Taphrinomycotina							
									Class Neolectomycetes				
													Order Neolectales
									Class Pneumocystidomycetes				
													Order Pneumocystidales
									Class Schizosaccharomycetes				
													Order Schizosaccharomycetales
									Class Taphrinomycetes				
													Order Taphrinales
					Phylum Basidiomycota								
									Class Entorrhizomycetes				
													Order Entorrhizales
													Order Wallemiales
						Subphylum Agaricomycotina							
									Class Agaricomycetes				
										Subclass N.N.			
													Order Auriculariales
													Order Cantharellales
													Order Corticiales
													Order Gloeophyllales
													Order Hymenochaetales
													Order Polyporales
													Order Russulales
													Order Sebacinales
													Order Thelephorales
													Order Trechisporales
										Subclass Agaricomycetidae			
													Order Agaricales
													Order Atheliales
													Order Boletales
										Subclass Phallomycetidae			
													Order Geastrales
													Order Gomphales
													Order Hysterangiales
													Order Phallales
									Class Dacrymycetes				
													Order Dacrymycetales
									Class Tremellomycetes				
													Order Cystofilobasidiales
													Order Filobasidiales
													Order Tremellales
						Subphylum Pucciniomycotina							
									Class Agaricostilbomycetes				
													Order Agaricostilbales
													Order Spiculogloeales
									Class Atractiellomycetes				
													Order Atractiellales
									Class Classiculomycetes				
													Order Classiculales
									Class Cryptomycocolacomycetes				
													Order Cryptomycocolacales
									Class Cystobasidiomycetes				
													Order Cystobasidiales
													Order Erythrobasidiales
													Order Naohideales
									Class Microbotryomycetes				
													Order Hetrogastridiales
													Order Leucosporidiales
													Order Microbotryales
													Order Sporidiobolales
									Class Mixiomycetes				
													Order Mixiales
									Class Pucciniomycetes				
													Order Helicobasidiales
													Order Pachnocybales
													Order Platygloeales
													Order Pucciniales
													Order Septobasidiales
						Subphylum Ustilaginomycotina							
									Class N.N.				
													Order Malasseziales
									Class Exobasidiomycetes				
													Order Ceraceosorales
													Order Doassansiales
													Order Entylomatales
													Order Exobasidiales
													Order Georgefischeriales
													Order Microstromatales
													Order Tilletiales
									Class Ustilaginomycetes				
													Order Urocystidales
													Order Ustilaginales
		SUBKINGDOM EOMYCOTA											
					Phylum Chytridiomycota								
									Class Blastocladiomycetes [= Allomycetes]				
													Order Blastocladiales
									Class Chytridiomycetes				
													Order Chytridiales
													Order Lobulomycetales
													Order Neocallimastigales
													Order Olpidiales
													Order Rhizophlyctidales
													Order Rhizophydiales
													Order Spizellomycetales
									Class Monoblepharidomycetes				
													Order Monoblepharidales
					Phylum Glomeromycota								
									Class Glomeromycetes [= Glomomycetes]				
													Order Archaeosporales
													Order Diversisporales
													Order Glomerales
													Order Paraglomerales
					Phylum Zygomycota								
						Subphylum N.N.							
									Class N.N.				
													Order Basidiobolales
						Subphylum Entomophthoromycotina							
									Class N.N.				
													Order Entomophthorales
						Subphylum Kickxellomycotina							
									Class N.N.				
													Order Asellariales
													Order Dimargaritales
													Order Harpellales
													Order Kickxellales
						Subphylum Mortierellomycotina							
									Class N.N.				
													Order Mortierellales
						Subphylum Mucoromycotina							
									Class N.N.				
													Order Endogonales
													Order Mucorales
						Subphylum Zoopagomycotina							
									Class N.N.				
													Order Zoopagales
	**KINGDOM PLANTAE**												
		SUBKINGDOM BILIPHYTA											
					Phylum Glaucophyta								
									Class Glaucophyceae				
													Order Glaucocystales
					Phylum Rhodophyta								
						Subphylum Cyanidiophytina							
									Class Cyanidiophyceae				
													Order Cyanidiales
						Subphylum Eurhodophytina							
									Class Bangiophyceae				
													Order Bangiales
													Order Goniotrichales
									Class Florideophyceae				
										Subclass N.N.			
													Order Rhodachlyales
										Subclass Ahnfeltiophycidae			
													Order Ahnfeltiales
													Order Pihiellales
										Subclass Corallinophycidae			
													Order Corallinales
													Order Rhodogorgonales
													Order Sporolithales
										Subclass Hildenbrandiophyceae			
													Order Hildenbrandiales
										Subclass Nemaliophycidae			
													Order Acrochaetiales
													Order Balbianiales
													Order Balliales
													Order Batrachospermales
													Order Colaconematales
													Order Entwisleiales
													Order Nemaliales
													Order Palmariales
													Order Thoreales
										Subclass Rhodymeniophycidae			
													Order Acrosymphytales
													Order Bonnemaisoniales
													Order Ceramiales
													Order Gelidiales
													Order Gigartinales
													Order Gracilariales
													Order Halymeniales
													Order Nemastomatales
													Order Peyssonneliales
													Order Plocamiales
													Order Rhodymeniales
													Order Sebdeniales
						Subphylum Metarhodophytina							
									Class Compsopogonophyceae				
													Order Compsopogonales
													Order Erythropeltidales
													Order Rhodochaetales
						Subphylum Rhodellophytina							
									Class Porphyridiophyceae				
													Order Porphyridiales
									Class Rhodellophyceae				
													Order Dixoniellales
													Order Glaucosphaerales
													Order Rhodellales
									Class Stylonematophyceae				
													Order Rufusiales
													Order Stylonematales
		SUBKINGDOM VIRIDIPLANTAE											
			INFRAKINGDOM CHLOROPHYTA										
					Phylum Chlorophyta								
						Subphylum Chlorophytina							
									Class Chlorodendrophyceae				
													Order Chlorodendrales
									Class Chlorophyceae				
													Order N.N. (e.g., Chlorangiopsidaceae)
													Order Chaetopeltidales
													Order Chaetophorales
													Order Chlamydomonadales [= Volvocales]
													Order Oedogoniales
													Order Sphaeropleales
									Class Pedinophyceae				
													Order Marsupiomonadales
													Order Pedinomonadales
													Order Scourfieldiales
									Class Trebouxiophyceae				
													Order Chlorellales
													Order Microthamniales
													Order Phyllsiphonales
													Order Prasiolales
													Order Trebouxiales
									Class Ulvophyceae				
													Order Bryopsidales
													Order Cladophorales
													Order Dasycladales
													Order Oltmansiellopsidales
													Order Scotinosphaerales
													Order Trentepohliales
													Order Ulotrichales
													Order Ulvales
						Subphylum Prasinophytina							
									Class Mamiellophyceae				
													Order Dolichomastigales
													Order Mamiellales
													Order Monomastigales
									Class Nephrophyceae [= Nephroselmidophyceae]				
													Order Nephroselmidales
									Class Pyramimonadophyceae				
													Order Palmophyllales
													Order Prasinococcales
													Order Pseudoscourfieldiales
													Order Pyramimonadales
			INFRAKINGDOM STREPTOPHYTA										
				Superphylum Charophyta									
					Phylum Charophyta								
									Class Charophyceae				
													Order Charales
									Class Chlorokybophyceae				
													Order Chlorokybales
									Class Coleochaetophyceae				
													Order Chaetosphaeridiales
													Order Coleochaetales
									Class Conjugatophyceae [= Zygnematophyceae]				
													Order Desmidiales
													Order Zygnematales
									Class Klebsormidiophyceae				
													Order Klebsormidiales
									Class Mesostigmatophyceae				
													Order Mesostigmatales
				Superphylum Embryophyta									
					Phylum Anthocerotophyta								
									Class Anthocerotopsida				
										Subclass Anthocerotidae			
													Order Anthocerotales
										Subclass Dendrocerotidae			
													Order Dendrocerotales
													Order Phymatocerales
										Subclass Notothylatidae			
													Order Notothyladales
									Class Leiosporocerotopsida				
													Order Leiosporocerotales
					Phylum Bryophyta								
									Class Andreaeobryopsida				
													Order Andreaeobryales
									Class Andreaeopsida				
													Order Andreaeales
									Class Bryopsida				
										Subclass Bryidae			
													Order Bartramiales
													Order Bryales
													Order Hedwigiales
													Order Hookeriales
													Order Hypnales
													Order Hypnodendrales
													Order Orthotrichales
													Order Ptychomniales
													Order Rhizogoniales
													Order Splachnales
										Subclass Buxbaumiidae			
													Order Buxbaumiales
										Subclass Dicranidae			
													Order Archidiales
													Order Bryoxiphiales
													Order Dicranales
													Order Grimmiales
													Order Pottiales
													Order Scouleriales
										Subclass Diphysciidae			
													Order Diphysciales
										Subclass Funariidae			
													Order Encalyptales
													Order Funariales
													Order Gigaspermales
										Subclass Timmidae			
													Order Timmiales
									Class Oedipodiopsida				
													Order Oedipodiales
									Class Polytrichopsida				
													Order Polytrichales
									Class Sphagnopsida				
													Order Ambuchananiales
													Order Sphagnales
									Class Takakiopsida				
													Order Takakiales
									Class Tetraphidopsida				
													Order Tetraphidales
					Phylum Marchantiophyta								
									Class Haplomitriopsida				
													Order Calobryales
													Order Treubiales
									Class Jungermanniopsida				
										Subclass Jungermanniidae			
													Order Jungermanniales
													Order Porellales
													Order Ptilidiales
										Subclass Metzgeriidae			
													Order Metzgeriales
													Order Pleuroziales
										Subclass Pelliidae			
													Order Fossombroniales
													Order Pallaviciniales
													Order Pelliales
									Class Marchantiopsida				
													Order Blasiales
													Order Lunulariales
													Order Marchantiales
													Order Neohodgsoniales
													Order Sphaerocarpales
					Phylum Tracheophyta								
						Subphylum Lycopodiophytina							
									Class Lycopodiopsida				
													Order Isoetales
													Order Lycopodiales
													Order Selaginellales
						Subphylum Polypodiophytina							
									Class Polypodiopsida				
										Subclass Equisetidae			
													Order Equisetales
										Subclass Marattiidae			
													Order Marattiales
										Subclass Ophioglossidae [= Psilotidae]			
													Order Ophioglossales
													Order Psilotales
										Subclass Polypodiidae			
													Order Cyatheales
													Order Gleicheniales
													Order Hymenophyllales
													Order Osmundales
													Order Polypodiales
													Order Salviniales
													Order Schizaeales
						Subphylum Spermatophytina							
								Superclass “Angiospermae”					
									Class Magnoliopsida				
												Superorder N.N.	
													Order N.N. (e.g., Icacinaceae)
												Superorder Amborellanae	
													Order Amborellales
												Superorder Asteranae	
													Order Apiales
													Order Aquifoliales
													Order Asterales
													Order Boraginales
													Order Bruniales
													Order Cornales
													Order Dipsacales
													Order Ericales
													Order Escalloniales
													Order Garryales
													Order Gentianales
													Order Lamiales
													Order Paracryphiales
													Order Solanales
												Superorder Austrobaileyanae	
													Order Austrobaileyales
												Superorder Berberidopsidanae	
													Order Berberidopsidales
												Superorder Buxanae	
													Order Buxales
												Superorder Caryophyllanae	
													Order Caryophyllales
												Superorder Ceratophyllanae	
													Order Ceratophyllales
												Superorder Dillenianae	
													Order Dilleniales
												Superorder Lilianae [= Monocotyledones]	
													Order Acorales
													Order Alismatales
													Order Arecales
													Order Asparagales
													Order Commelinales
													Order Dasypogonales
													Order Dioscoreales
													Order Liliales
													Order Pandanales
													Order Petrosaviales
													Order Poales
													Order Zingiberales
												Superorder Magnolianae	
													Order Canellales
													Order Chloranthales
													Order Laurales
													Order Magnoliales
													Order Piperales
												Superorder Myrothamnanae	
													Order Gunnerales
												Superorder Nymphaeanae	
													Order Nymphaeales
												Superorder Proteanae	
													Order Proteales
												Superorder Ranunculanae	
													Order Ranunculales
												Superorder Rosanae	
													Order Brassicales
													Order Celastrales
													Order Crossosomatales
													Order Cucurbitales
													Order Fabales
													Order Fagales
													Order Geraniales
													Order Huerteales
													Order Malpighiales
													Order Malvales
													Order Myrtales
													Order Oxalidales
													Order Picramniales
													Order Rosales
													Order Sapindales
													Order Vitales
													Order Zygophyllales
												Superorder Santalanae	
													Order Santalales
												Superorder Saxifraganae	
													Order Saxifragales
												Superorder Trochodendranae	
													Order Trochodendrales
								Superclass “Gymnospermae”					
									Class Cycadopsida				
										Subclass Cycadidae			
													Order Cycadales
									Class Ginkgoopsida				
										Subclass Ginkgooidae			
													Order Ginkgoales
									Class Gnetopsida				
										Subclass Gnetidae			
													Order Gnetales
									Class Pinopsida				
										Subclass Pinidae			
													Order Pinales
	**KINGDOM ANIMALIA**												
		SUBKINGDOM N.N.											
					Phylum Cnidaria								
						Subphylum Anthozoa							
									Class Anthozoa				
										Subclass Hexacorallia			
													Order Actiniaria
													Order Antipatharia
													Order Ceriantharia
													Order Corallimorpharia
													Order Scleractinia
													Order Zoantharia [= Zoanthidea]
										Subclass Octocorallia			
													Order Alcyonacea
													Order Helioporacea
													Order Pennatulacea
						Subphylum Medusozoa							
									Class Cubozoa				
													Order Carybdeida
													Order Chirodropida
									Class Hydrozoa				
										Subclass Hydroidolina			
													Order Anthoathecata
													Order Gonoproxima
													Order Leptothecata
													Order Siphonophorae
										Subclass Trachylina			
													Order Actinulida
													Order Limnomedusae
													Order Narcomedusae
													Order Trachymedusae
									Class Polypodiozoa				
													Order Polypodiidea
									Class Scyphozoa				
													Order Coronatae
													Order Rhizostomeae
													Order Semaeostomeae
									Class Staurozoa				
													Order Stauromedusae
						Subphylum Myxozoa							
									Class Malacosporea				
													Order Malacovalvulida
									Class Myxosporea				
													Order Bivalvulida
													Order Multivalvulida
					Phylum Ctenophora								
									Class Nuda				
													Order Beroida
									Class Tentaculata				
													Order Cambojiida
													Order Cestida
													Order Cryptolobiferida
													Order Cydippida
													Order Ganeshida
													Order Lobata
													Order Platyctenida
													Order Thalassocalycida
					Phylum Placozoa								
									Class Placozoa *(Trichoplax*)				
					Phylum Porifera								
									Class Calcarea				
													Order Baerida
													Order Clathrinida
													Order Leucosolenida
													Order Lithonida
													Order Murrayonida
									Class Demospongiae				
													Order Agelasida
													Order Astrophorida
													Order Chondrosida
													Order Dendroceratida
													Order Dictyoceratida
													Order Hadromerida
													Order Halichondrida
													Order Haplosclerida
													Order Lithistida
													Order Poecilosclerida
													Order Spirophorida
													Order Verongida
									Class Hexactinellida				
													Order Amphidiscosida
													Order Aulocalycoida
													Order Fieldingida
													Order Hexactinosida
													Order Lychniscosida
													Order Lyssacinosida
									Class Homoscleromorpha				
													Order Homosclerophorida
		SUBKINGDOM BILATERIA											
			INFRAKINGDOM PROTOSTOMIA										
				Superphylum N.N.									
					Phylum Chaetognatha								
									Class Sagittoidea				
													Order Aphragmophora
													Order Phragmophora
					Phylum Orthonectida								
													Order Plasmodigenea
					Phylum Rhombozoa								
													Order Dicyemida
													Order Heterocyemida
				Superphylum Ecdysozoa									
					Phylum Arthropoda								
						Subphylum Chelicerata							
									Class Arachnida				
												Superorder N.N.	
													Order Amblypygi
													Order Araneae
													Order Opiliones
													Order Palpigradi
													Order Pseudoscorpiones
													Order Ricinulei
													Order Schizomida
													Order Scorpiones
													Order Solifugae
													Order Uropygi
												Superorder Acariformes	
													Order Sarcoptiformes
													Order Trombidiformes
												Superorder Parasitiformes	
													Order Holothyrida
													Order Ixodida
													Order Mesostigmata
													Order Opilioacarida
									Class Merostomata				
													Order Xiphosura
									Class Pycnogonida				
													Order Pantopoda
						Subphylum Crustacea							
									Class Branchiopoda				
													Order Anostraca
													Order Diplostraca
													Order Laevicaudata
													Order Notostraca
									Class Cephalocarida				
													Order Brachypoda
									Class Malacostraca				
										Subclass Eumalacostraca			
												Superorder Eucarida	
													Order Amphionidacea
													Order Decapoda
													Order Euphausiacea
												Superorder Peracarida	
													Order Amphipoda
													Order Bochusacea
													Order Cumacea
													Order Isopoda
													Order Lophogastrida
													Order Mictacea
													Order Mysida
													Order Spelaeogriphacea
													Order Tanaidacea
													Order Thermosbaenacea
												Superorder Syncarida	
													Order Anaspidacea
													Order Bathynellacea
										Subclass Hoplocarida			
													Order Stomatopoda
										Subclass Phyllocarida			
													Order Leptostraca
									Class Maxillopoda				
										Subclass Branchiura			
													Order Arguloida
										Subclass Copepoda			
											Infraclass Neocopepoda		
												Superorder Gymnoplea	
													Order Calanoida
												Superorder Podoplea	
													Order Cyclopoida
													Order Gelyelloida
													Order Harpacticoida
													Order Misophrioida
													Order Monstrilloida
													Order Mormonilloida
													Order Siphonostomatoida
											Infraclass Progymnoplea		
													Order Platycopioida
										Subclass Mystacocarida			
													Order Mystacocaridida
										Subclass Pentastomida			
													Order Cephalobaenida
													Order Porocephalida
										Subclass Tantulocarida (e.g., Basipodellidae)			
										Subclass Thecostraca			
											Infraclass Ascothoracida		
													Order Dendrogastrida
													Order Laurida
											Infraclass Cirripedia		
												Superorder Acrothoracica	
													Order Cryptophialida
													Order Lithoglyptida
												Superorder Rhizocephala	
													Order Akentrogonida
													Order Kentrogonida
												Superorder Thoracica	
													Order Ibliformes
													Order Lepadiformes
													Order Scalpelliformes
													Order Sessilia
											Infraclass Facetotecta (*Hansenocaris*)		
									Class Ostracoda				
													Order Halocyprida
													Order Myodocopida
													Order Paleocopida
													Order Platycopida
													Order Podocopida
									Class Remipedia				
													Order Nectiopoda
						Subphylum Hexapoda							
									Class Collembola				
													Order Entomobryomorpha
													Order Neelipleona
													Order Poduromorpha
													Order Symphypleona
									Class Diplura				
													Order N.N. (e.g., *Japygidae*)
									Class Insecta				
										Subclass Archaeognatha			
													Order Archaeognatha
										Subclass Dicondylia			
													Order Zygentoma
										Subclass Pterygota			
											Infraclass Neoptera		
												Superorder Holometabola	
													Order Coleoptera
													Order Diptera
													Order Hymenoptera
													Order Lepidoptera
													Order Mecoptera
													Order Siphonaptera
													Order Strepsiptera
													Order Trichoptera
												Superorder Neuropterida	
													Order Megaloptera
													Order Neuroptera
													Order Raphidioptera
												Superorder Paraneoptera	
													Order Hemiptera
													Order Psocodea
													Order Thysanoptera
												Superorder Polyneoptera	
													Order Blattodea
													Order Dermaptera
													Order Embioptera
													Order Grylloblattodea
													Order Mantodea
													Order Mantophasmatodea
													Order Orthoptera
													Order Phasmida
													Order Plecoptera
													Order Zoraptera
											Infraclass Palaeoptera		
													Order Ephemeroptera
													Order Odonata
									Class Protura				
													Order Acerentomata
													Order Eosentomata
													Order Sinentomata
						Subphylum Myriapoda							
									Class Chilopoda				
													Order Craterostigmomorpha
													Order Geophilomorpha
													Order Lithobiomorpha
													Order Scolopendromorpha
													Order Scutigeromorpha
									Class Diplopoda				
										Subclass Chilognatha			
											Infraclass Helminthomorpha		
												Superorder N.N.	
													Order Platydesmida
													Order Polyzoniida
													Order Siphonocryptida
													Order Siphonophorida
												Superorder Juliformia	
													Order Julida
													Order Spirobolida
													Order Spirostreptida
												Superorder Nematophora	
													Order Callipodida
													Order Chordeumatida
													Order Stemmiulida
													Order Siphoniulida
												Superorder Merochaeta	
													Order Polydesmida
											Infraclass Pentazonia		
													Order Glomerida
													Order Glomeridesmida
													Order Sphaerotheriida
										Subclass Penicillata			
													Order Polyxenida
									Class Pauropoda				
													Order Hexamerocerata
													Order Tetramerocerata
									Class Symphyla (e.g., Scolopendrellidae)				
					Phylum Kinorhyncha								
													Order Cyclorhagida
													Order Homalorhagida
					Phylum Loricifera								
													Order Nanaloricida
					Phylum Nematoda								
									Class Chromadorea				
										Subclass Chromadoria			
													Order Chromadorida
													Order Desmodorida
													Order Desmoscolecida
													Order Selachinematida
										Subclass Plectia			
												Superorder Monhysterica	
													Order Monhysterida
												Superorder Plectica	
													Order Benthimermithida
													Order Leptolaimida
													Order Plectida
												Superorder Rhabditica	
													Order Diplogasterida
													Order Drilonematida
													Order Panagrolaimida
													Order Rhabditida
													Order Spirurida
												Superorder Teratocephalica	
													Order Teratocephalida
									Class Dorylaimea				
										Subclass Bathyodontia			
													Order Bathyodontida
													Order Mermithida
													Order Mononchida
										Subclass Dorylaimia			
													Order Dorylaimida
										Subclass Trichocephalia			
													Order Dioctophymatida
													Order Marimermithida
													Order Muspiceida
													Order Trichocephalida
									Class Enoplea				
										Subclass Enoplia			
													Order Alaimida
													Order Enoplida
													Order Ironida
													Order Rhaptothyreida
													Order Trifusiida
													Order Tripyloidida
										Subclass Oncholaimia			
													Order Oncholaimida
										Subclass Triplonchia			
													Order Triplonchida
													Order Tripylida
					Phylum Nematomorpha								
													Order Gordioidea
													Order Nectonematoidea
					Phylum Onychophora								
									Class Udeonycophora				
													Order Euonycophora
					Phylum Priapula [= Priapulida]								
									Class N.N. (e.g., Priapulidae)				
					Phylum Tardigrada								
									Class Eutardigrada				
													Order Apochela
													Order Parachela
									Class Heterotardigrada				
													Order Arthrotardigrada
													Order Echiniscoidea
				Superphylum Spiralia [= Lophotrochozoa]									
					Phylum Acanthocephala								
									Class Archiacanthocephala				
													Order Apororhynchida
													Order Gigantorhynchida
													Order Moniliformida
													Order Oligacanthorhynchida
									Class Eoacanthocephala				
													Order Gyracanthocephala
													Order Neoechinorhynchida
									Class Palaeacanthocephala				
													Order Echinorhynchida
													Order Heteramorphida
													Order Polymorphida
									Class Polyacanthocephala				
													Order Polyacanthorhynchida
					Phylum Annelida								
									Class N.N.				
													Order Myzostomida
									Class Clitellata				
										Subclass N.N.			
													Order Branchiobdellida
										Subclass Hirudinea			
													Order Acanthobdellida
													Order Arhynchobdellida
													Order Rhynchobdellida
										Subclass Oligochaeta			
												Superorder N.N.	
													Order N.N. (*Jennaria*)
													Order Enchytraeida
													Order Haplotaxida
													Order Lumbriculida
													Order Tubificida
												Superorder Metagynophora	
													Order Moniligastrida
													Order Opistophora
									Class Polychaeta				
										Subclass N.N.			
													Order N.N. (e.g., Nerillidae)
										Subclass Echiura			
													Order Echiuroinea
													Order Heteromyota
													Order Xenopneusta
										Subclass Errantia			
													Order Amphinomida
													Order Eunicida
													Order Phyllodocida
										Subclass Sedentaria			
											Infraclass Canalipalpata		
													Order Sabellida
													Order Spionida
													Order Terebellida
											Infraclass Scolecida (e.g., Arenicolidae)		
					Phylum Brachiopoda								
									Class Craniata				
													Order Craniida
									Class Lingulata				
													Order Lingulida
									Class Rhynchonellata				
													Order Rhynchonellida
													Order Terebratulida
													Order Thecideida
					Phylum Bryozoa								
									Class Gymnolaemata				
													Order Cheilostomata
													Order Ctenostomata
									Class Phylactolaemata				
													Order Plumatellida
									Class Stenolaemata				
													Order Cyclostomata
					Phylum Cycliophora								
									Class Eucycliophora				
													Order Symbiida
					Phylum Entoprocta								
													Order Coloniales
													Order Solitaria
					Phylum Gastrotricha								
													Order Chaetonotida
													Order Macrodasyida
					Phylum Gnathostomulida								
													Order Bursovaginoidea
													Order Filospermoidea
					Phylum Micrognathozoa								
									Class Micrognathozoa				
													Order Limnognathida
					Phylum Mollusca								
									Class Bivalvia				
										Subclass Autobranchia			
												Superorder Heteroconchia	
													Order Carditida
													Order Lucinida
													Order Myida
													Order Pholadomyida
													Order Trigoniida
													Order Unionida
													Order Veneroida
												Superorder Pteriomorphia	
													Order Arcida
													Order Limida
													Order Mytilida
													Order Ostreida
													Order Pectinida
													Order Pteriida
										Subclass Protobranchia			
													Order Nuculanida
													Order Nuculida
													Order Solemyoida
									Class Caudofoveata				
													Order Chaetodermatida
									Class Cephalopoda				
										Subclass Coleoidea			
												Superorder Decabrachia	
													Order Sepiida
													Order Sepiolida
													Order Spirulida
													Order Teuthida
												Superorder Octobrachia	
													Order Octopoda
													Order Vampyromorphida
										Subclass Nautiloidea			
													Order Nautilida
									Class Gastropoda				
										Subclass Caenogastropoda			
													Order Littorinimorpha
													Order Neogastropoda
										Subclass Cocculiniformia (e.g., Cocculinidae)			
										Subclass Heterobranchia			
													Order Acochlidioidea
													Order Anaspidea
													Order Cephalaspidea
													Order Gymnosomata
													Order Hygrophila
													Order Nudibranchia
													Order Pleurobranchomorpha
													Order Runcinacea
													Order Sacoglossa
													Order Stylommatophora
													Order Systellommatophora
													Order Thecosomata
													Order Umbraculida
										Subclass Neomphalina			
													Order N.N. (e.g., Neomphalidae)
										Subclass Neritimorpha			
													Order Cycloneritimorpha
										Subclass Patellogastropoda			
													Order N.N. (e.g., Patellidae)
										Subclass Vetigastropoda			
													Order N.N. (e.g., Ataphridae)
									Class Monoplacophora				
													Order Tryblidiida
									Class Polyplacophora				
													Order Chitonida
													Order Lepidopleurida
									Class Scaphopoda				
													Order Dentaliida
													Order Gadilida
									Class Solenogastres				
												Superorder Aplotegmentaria	
													Order Cavibelonia
													Order Sterrofustia
												Superorder Pachytegmentaria	
													Order Neomeniamorpha
													Order Pholidoskepia
					Phylum Nemertea								
									Class Anopla				
													Order N.N. (e.g., Gorgonorhynchidae)
									Class Enopla				
													Order Monostilifera
													Order Polystilifera
									Class Paleonemertea (e.g., Carinomidae)				
					Phylum Phoronida								
									Class N.N. (e.g., *Phoronis*)				
					Phylum Platyhelminthes								
						Subphylum Catenulidea							
													Order Catenulida
						Subphylum Rhabditophora							
									Class Macrostomorpha				
													Order Haplopharyngida
													Order Macrostomida
									Class Neoophora				
										Subclass Eulecithophora			
											Infraclass Adiaphanida		
													Order Fecampiida
													Order Prolecithophora
													Order Tricladida
											Infraclass Rhabdocoela		
													Order Dalytyphloplanida
													Order Endoaxonemata
													Order Kalyptorhynchia
										Subclass Neodermata			
											Infraclass Cestoda		
													Order Amphilinidea
													Order Bothriocephalidea
													Order Caryophyllidea
													Order Cyclophyllidea
													Order Diphyllidea
													Order Diphyllobothriidea
													Order Gyrocotylidea
													Order Lecanicephalidea
													Order Litobothridea
													Order Proteocephalidea
													Order Pseudophyllidea
													Order Rhinebothriidea
													Order Spathebothriidea
													Order Tetrabothriidea
													Order Tetraphyllidea
													Order Trypanorhyncha
											Infraclass Monogenea		
													Order Capsalidea
													Order Chimaericolidea
													Order Dactylogyridea
													Order Diclybothriidea
													Order Gyrodactylidea
													Order Mazocraeidea
													Order Monocotylidea
													Order Montchadskyellidea
													Order Polystomatidea
											Infraclass Trematoda		
													Order Aspidogastrida
													Order Diplostomida
													Order Plagiorchiida
													Order Stichocotylida
									Class Polycladidea				
													Order Lecithoepitheliata
													Order Polycladida
										Subclass Proseriatia			
													Order Proseriata
					Phylum Rotifera								
									Class Eurotatoria				
										Subclass Bdelloidea (e.g., Adinetidae)			
										Subclass Monogonta			
													Order Collothecaceae
													Order Flosculariaceae
													Order Ploima
									Class Pararotatoria				
													Order Seisonacea
					Phylum Sipuncula								
									Class Phascolosomatidea				
													Order Aspidosiphoniformes
													Order Phascolosomatiformes
									Class Sipunculidea				
													Order Golfingiiformes
													Order Sipunculiformes
			INFRAKINGDOM DEUTEROSTOMIA										
					Phylum Chordata								
						Subphylum Cephalochordata							
													Order Amphioxiformes
						Subphylum Urochordata							
									Class Appendicularia				
													Order Copelata
									Class Ascidiacea				
													Order Enterogona
													Order Pleurogona
									Class Thaliacea				
													Order Doliolida
													Order Pyrosomida
													Order Salpida
						Subphylum Vertebrata [= Craniata]							
							Infraphylum Agnatha						
									Class Cephalaspidomorphi				
													Order Petromyzontiformes
									Class Myxini				
													Order Myxiniformes
							Infraphylum Gnathostomata						
								Superclass Actinopterygii					
									Class Chondrostei				
													Order Acipenseriformes
									Class Cladistei				
													Order Polypteriformes
									Class Holostei				
													Order Amiiformes
													Order Lepisosteiformes
									Class Teleostei				
													Order Acanthuriformes
													Order Albuliformes
													Order Alepocephaliformes
													Order Anabantiformes
													Order Anguilliformes
													Order Argentiniformes
													Order Ateleopodiformes
													Order Atheriniformes
													Order Aulopiformes
													Order Batrachoidiformes
													Order Beloniformes
													Order Beryciformes
													Order Blenniiformes
													Order Carangiformes
													Order Centrarchiformes
													Order Characiformes
													Order Cichliformes
													Order Cirrhitiformes
													Order Clupeiformes
													Order Cypriniformes
													Order Cyprinodontiformes
													Order Elopiformes
													Order Ephippiformes
													Order Esociformes
													Order Gadiformes
													Order Galaxiiformes
													Order Gobiiformes
													Order Gonorynchiformes
													Order Gymnotiformes
													Order Hidontiformes
													Order Holocentriformes
													Order Istiophoriformes
													Order Kurtiformes
													Order Labriformes
													Order Lampridiformes
													Order Lepidogalaxiiformes
													Order Lobotiformes
													Order Lophiiformes
													Order Mugiliformes
													Order Myctophiformes
													Order Notacanthiformes
													Order Ophidiiformes
													Order Osmeriformes
													Order Osteoglossiformes
													Order Pempheriformes
													Order Perciformes
													Order Percopsiformes
													Order Pholidichthyiformes
													Order Pleuronectiformes
													Order Polymixiiformes
													Order Salmoniformes
													Order Scombriformes
													Order Siluriformes
													Order Spariformes
													Order Stomiatiformes
													Order Stylephoriformes
													Order Synbranchiformes
													Order Syngnathiformes
													Order Terapontiformes
													Order Tetraodontiformes
													Order Uranoscopiformes
													Order Zeiformes
								Superclass Chondrichthyes					
									Class Elasmobranchii				
													Order Carcharhiniformes
													Order Heterodontiformes
													Order Hexanchiformes
													Order Lamniformes
													Order Myliobatiformes
													Order Orectolobiformes
													Order Pristiformes
													Order Pristiophoriformes
													Order Rajiformes
													Order Squaliformes
													Order Squatiniformes
													Order Torpediniformes
									Class Holocephali				
													Order Chimaeriformes
								Superclass Sarcopterygii					
									Class Coelacanthi				
													Order Coelacanthiformes
									Class Dipnoi				
													Order Ceratodontiformes
													Order Lepidosirenoformes
								Superclass Tetrapoda					
									Class Amphibia				
													Order Anura
													Order Caudata
													Order Gymnophiona
									Class Mammalia				
										Subclass Prototheria			
													Order Monotremata
										Subclass Theria			
											Infraclass Eutheria [= Placentalia]		
													Order Afrosoricida
													Order Artiodactyla
													Order Carnivora
													Order Cetacea
													Order Chiroptera
													Order Cingulata
													Order Dermoptera
													Order Erinaceomorpha
													Order Hyracoidea
													Order Lagomorpha
													Order Macroscelidea
													Order Perissodactyla
													Order Pholidota
													Order Pilosa
													Order Primates
													Order Proboscidea
													Order Rodentia
													Order Scandentia
													Order Sirenia
													Order Soricomorpha
													Order Tubulidentata
											Infraclass Metatheria [= Marsupialia]		
													Order Dasyuromorphia
													Order Didelphimorphia
													Order Diprotodontia
													Order Microbiotheria
													Order Notoryctemorphia
													Order Paucituberculata
													Order Peramelemorphia
									Class Reptilia				
										Subclass Aves			
											Infraclass Neognathae		
												Superorder Galloanseri	
													Order Anseriformes
													Order Galliformes
												Superorder Neoaves	
													Order Accipitriformes
													Order Apodiformes
													Order Bucerotiformes
													Order Caprimulgiformes
													Order Cariamiformes
													Order Charadriiformes
													Order Ciconiiformes
													Order Coliiformes
													Order Columbiformes
													Order Coraciiformes
													Order Cuculiformes
													Order Eurypygiformes
													Order Falconiformes
													Order Gaviiformes
													Order Gruiformes
													Order Leptosomiformes
													Order Mesitornithiformes
													Order Musophagiformes
													Order Opisthocomiformes
													Order Otidiformes
													Order Passeriformes
													Order Pelecaniformes
													Order Phaethontiformes
													Order Phoenicopteriformes
													Order Piciformes
													Order Podicipediformes
													Order Procellariiformes
													Order Psittaciformes
													Order Pteroclidiformes
													Order Sphenisciformes
													Order Strigiformes
													Order Suliformes
													Order Trogoniformes
											Infraclass Paleognathae		
													Order Apterygiformes
													Order Casuariiformes
													Order Rheiformes
													Order Struthioniformes
													Order Tinamiformes
										Subclass Crocodylomorpha			
													Order Crocodylia
										Subclass Rhynchocephalia			
													Order Sphenodontida
										Subclass Squamata			
													Order Anguimorpha
													Order Gekkota
													Order Inguania
													Order Lacertoidea
													Order Scincoidea
													Order Serpentes
										Subclass Testudinata			
													Order Testudines
					Phylum Echinodermata								
						Subphylum Asterozoa							
									Class Asteroidea				
													Order Brisingida
													Order Forcipulatida
													Order Notomyotida
													Order Paxillosida
													Order Peripoda
													Order Spinulosida
													Order Valvatida
													Order Velatida
									Class Ophiuroidea				
													Order Euryalida
													Order Ophiurida
						Subphylum Crinozoa							
									Class Crinoidea				
													Order Comatulida
													Order Cyrtocrinida
													Order Hyocrinida
													Order Isocrinida
						Subphylum Echinozoa							
									Class Echinoidea				
										Subclass Cidaroidea			
													Order Cidaroida
										Subclass Euechinoidea			
											Infraclass N.N.		
													Order Echinothurioida
											Infraclass Acroechinoidea		
													Order Aspidodiadematoida
													Order Diadematoida
													Order Micropygoida
													Order Pedinoida
											Infraclass Carinacea		
													Order Arbacioida
													Order Camarodonta
													Order Salenioida
													Order Stomopneustoida
											Infraclass Irregularia		
													Order Cassiduloida
													Order Clypeasteroida
													Order Echinolampadoida
													Order Holasteroida
													Order Spatangoida
									Class Holothuroidea				
													Order N.N. (*Thyone*)
													Order Apodida
													Order Aspidochirotida
													Order Dendrochirotida
													Order Elasipodida
													Order Molpadida
					Phylum Hemichordata								
									Class Enteropneusta (e.g., Harrimaniidae)				
									Class Pterobranchia				
										Subclass Cephalodiscida (*Cephalodiscus*)			
										Subclass Graptolithina			
													Order Rhabdopleurida
					Phylum Xenacoelomorpha								
						Subphylum Acoelomorpha							
									Class Acoela (e.g., Diopisthoporidae)				
									Class Nemertodermatida (e.g., Nemertodermatidae)				
						Subphylum Xenoturbellida							
									Class N.N. (Xenoturbellidae)				

Names below rank of infrakingdom are arranged alphabetically within each parent rank, except for taxa that are not named (N.N.). Brackets indicate synonyms. Quoted names are not validly published but in common use.

## Supporting Information

S1 Table Proposed hierarchical classification from superkingdom to order. (XLSX)
