# Supplementary material for: A Higher Level Classification of All Living Organisms
Source: PLoS One. 2015 Apr 29;10(4):e0119248. doi: 10.1371/journal.pone.0119248 (PMC4418965; doi:10.1371/journal.pone.0119248)
Supplement: S1 Appendix — (PDF) [file pone.0119248.s001.pdf]

S1 Appendix. List of sources consulted for proposed higher level classification of all living organisms.

Adl SM, Simpson AGB, Farmer MA, Andersen RA, Anderson OR, Barta JR, Bowser SS, Brugerolle G, Fensome RA, Fredericq S, James TY, Karpov S, Kugrens P, Krug J, Lane CE, Lewis LA, Lodge J, Lynn,DH, Mann DG, McCourt RM, Mendoza L, Moestrup Ø, Mozley-Standridge SE, Nerad TA, Shearer CA, Smirnov AV, Spiegel FW, Taylor MFJR. The new higher level classification of eukaryotes with emphasis on the taxonomy of protists. J Eukaryot Microbiol. 2005; 52: 399-451.

Adl SM, Simpson AGB, Lane CE, Lukes J, Bass D, Bowser SS, Brown MW, Burki F, Dunthorn M, Hampl V, Heiss A, Hoppenrath M, Lara E, Gall LL, Lynn DH, McManus H, Mitchell EAD, Mozley-Stanridge SE, Parfrey LW, Pawlowski J, Rueckert S, Shadwick L, Schoch C L, Smirnov A, Spiegel FW. The revised classification of eukaryotes. J Eukaryot Microbiol. 2012; 59: 429-493.

Ahyong ST, Lowry JK, Alonso M, Bamber RN, Boxshall GA, Castro P, Gerken S, Karaman GS, Goy JW, Jones DS, Meland K, Rogers DC, Svavarsson J. Subphylum Crustacea Brünnich, 1772. In: Zhang, Z-Q, editor. Animal biodiversity: an outline of higher-level classification and survey of taxonomic richness. Zootaxa. 2011; 3148: 165-191.

Amin O. Classification. In: Crompton DWT, Nickol B, editors. Biology of the Acanthocephala. London: Cambridge University Press; 1985.

Amin O. Key to the families and subfamilies of Acanthocephala with the erection of a new class (Polyacanthocephala) and a new order (Polyacanthorhynchida). J Parasitol. 1987; 73: 1216-1219.

Antunes A, Rainey FA, Wanner G, Taborda M, Pätzold J, Nobre MF, Da Costa MS, Huber R. A new lineage of halophilic, wall-less, contractile bacteria from a brine-filled deep of the Red Sea. J Bacteriol. 2008; 190: 3580-3587.

Bieler R, Carter J, Coan E. Classification of bivalve families. In: Bouchet P, Rocroi J-P, Bieler R, Carter J, Coan E. Nomenclator of bivalve families and classification of bivalve families. Malacologia. 2010; 52(2): 113-133.

Betancur-R R, Wiley EO, Miya M, Lecointre G, Bailly N, Ortí G. New and revised classification of bony fishes version 2; 2013. Available: http://deepfin.org/Classification_v2.htm. Accessed 01 April 2014.

Betancur-R R, Broughton RE, Wiley EO, Carpenter K, Lopez JA, Li C, Holcroft NI, Arcila D, Sanciangco M, Cureton J, Zhang F, Buser T, Campbell M, Rowley T, Ballesteros JA, Lu G, Grande T, Arratia G, Ortí G. The tree of life and a new classification of bony fishes. PLoS

Currents Tree of Life; 2013 Apr 18. Available: http://currents.plos.org/treeoflife/article/the-tree-of-life-and-a-new-classification-of-bony-fishes/.

Blackburn DC, Wake DB. Class Amphibia Gray, 1825. In: Zhang Z-Q, editor. Animal biodiversity: an outline of higher-level classification and survey of taxonomic richness. Zootaxa. 2011; 3148: 39-55.

Bock P, editor. Systematic list of families of Bryozoa; 2014. Available: http://bryozoa.net/famsys.html. Accessed 20 April 2014.

Bock P, Gordon D. Phylum Bryozoa Ehrenburg, 1831. In: Zhang Z-Q, editor. Animal biodiversity: an outline of higher-level classification and survey of taxonomic richness (Addenda 2013). Zootaxa. 2013; 3703: 67-74.

Bouchet P, Rocroi J, Fryda J, Hausdorf B, Ponder W, Valdes A, Waren A. Classification and nomenclator of gastropod families. Malacologia. 2005; 47(1-2): 1-397.

Bourlat SJ, Juliusdottir T, Lowe CJ, Freeman R, Aronowicz J, Kirschner M, Lander ES, Thorndyke M, Nakano H, Kohn AB, Heyland A, Moroz LL, Copley RR, Telford MJ. Deuterostome phylogeny reveals monophyletic chordates and the new phylum Xenoturbellida. Nature. 2006; 444: 85-88.

Brusca RC, Brusca GJ. Invertebrates, second edition. Sunderland, MA: Sinauer Assoc., Inc.; 2003.

Buckeridge J, Newman W. A revision of the Iblidae and the stalked barnacles (Crustacea: Cirripedia: Thoracica), including new ordinal, familial and generic taxa, and two new species from New Zealand and Tasmanian waters. Zootaxa. 2006; 1136: 1-38.

Capella-Gutiérrez S, Marcet-Houben M, Gabaldón T. Phylogenomics supports microsporidia as the earliest diverging clade of sequenced fungi. BMC Biology. 2012; 10: 47. Available: http://www.biomedcentral.com/1741-7007/10/47.

Cavalier-Smith T. A revised six-kingdom system of life. Biological Reviews. 1998; 73: 203-266.

Cavalier-Smith T. What are Fungi? In: McLaughlin DJ, McLaughlin EJ, Lemke P, editors. The Mycota, volume VII, Part A. Berlin: Springer-Verlag; 2000.

Cavalier-Smith T. The neomuran origin of archaebacteria, the negibacterial root of the universal tree and bacterial megaclassification. Int J Syst Evol Microbiol. 2002; 52: 7-76.

Cavalier-Smith T. The excavate protozoan phyla Metamonada Grassé emend. (Anaeromonadea, Parabasalia, Carpediemonas, Eopharyngia) and Loukozoa emend. (Jakobea, Malawimonas): their evolutionary affinities and new higher taxa. Int J Syst Evol Microbiol. 2003; 53: 1741-1758.

Cavalier-Smith T. Rooting the tree of life by transition analyses. Biology Direct. 2006; 1:19. doi:10.1186/1745-6150-1-19.

Cavalier-Smith T. Evolution and relationships of algae: major branches of the tree of life. In: Brodie J, Lewis J, editors. Unravelling the algae: the past, present and future. CRC Press; 2007. p. 21-56.

Cavalier-Smith T. Deep phylogeny, ancestral groups, and the four ages of life. Philos Trans R Soc Lond B Biol Sci. 2010; 365: 111-132.

Cavalier-Smith T. Kingdoms Protozoa and Chromista and the eozoan root of the eukaryotic tree. Biol Lett. 2010; 6: 342-345.

Cavalier-Smith T. Early evolution of eukaryote feeding modes, cell structural diversity, and classification of the protozoan phyla Loukozoa, Sulcozoa, and Choanozoa. Eur J Protistol. 2013; 49: 115-178.

Cavalier-Smith T, Scoble JM. Phylogeny of Heterokonta: Incisomonas marina, a uniciliate gliding opalozoan related to Solenicola (Nanomonadea), and evidence that Actinophryida evolved from raphidophytes. Eur J Protistol. 2013; 49 (3): 328-353.

Cavalier-Smith T, Fiore-Donno AM, Chao E, Kudryavtsev A, Berney C, Snell EA, Lewis R. Multigene phylogeny resolves deep branching of Amoebozoa. Mol Phylogenet Evol. 2014. http://dx.doi.org/10.1016/j.ympev.2014.08.011.

Chase MW, Reveal JL. A phylogenetic classification of the land plants to accompany APG III. Bot J Linn Soc. 2009; 161: 122-127.

Christenhusz MJM, Zhang X-C, Schneider H. A linear sequence of extant families and genera of lycophytes and ferns. Phytotaxa. 2011; 19: 7-54.

Christenhusz MJM, Reveal JL, Farjon A, Gardner MF, Mill RR, Chase MW. A new classification and linear sequence of extant gymnosperms. Phytotaxa. 2011; 19: 55-70.

Collins AG. Recent insights into cnidarian phylogeny. Smithsonian Contributions to the Marine Sciences. 2009; 38: 139-148.

Corradi N, Keeling PJ. Microsporidia: a journey through radical taxonomical revisions. Fungal Biol Rev. 2009; 23: 1-8.

Crandall-Stotler B, Stotler R, Long D. Phylogeny and classification of the Marchantiophyta. Edinburgh J Bot. 2009; 66: 155-198.

Crandall-Stotler B, Stotler R, Long D. Morphology and classification of the Marchantiophyta. In: Goffinet B, Shaw A. Bryophyte biology, 2nd Edition. Cambridge: Cambridge University Press; 2008.

Cronquist A. An integrated system of classification of flowering plants. New York: Columbia University Press; 1981.

Crowther AL. Class Anthozoa Ehrenberg, 1834. In: Zhang, Z-Q, editor. Animal biodiversity: an outline of higher-level classification and survey of taxonomic richness. Zootaxa. 2011; 3148: 19-23.

Cutler EB. The Sipuncula: Their systematics, biology, and evolution. Ithaca: Cornell University; 1994.

Daly M, Brugler MR, Cartwright P, Collins AG, Dawson MN, Fautin DG, France SC, Catherine P, McFadden S, Opresko DM, Rodriguez E, Romano SL, Stake JL. The phylum Cnidaria: a review of phylogenetic patterns and diversity 300 years after Linnaeus. Zootaxa. 2007; 1668: 127-182.

DeLey P, Blaxter M. A new system for Nematoda: combining morphological characters with molecular trees, and translating clades into ranks and taxa. Nematology Monographs and Perspectives. 2004; 2: 633-653.

Earle CJ. The Gymnosperm Database; 2013. Available: http://www.conifers.org/topics/sitemap.htm.

Emig CJ, Alvarez F, Bitner M. Brachiopoda World Database; 2013. Available at http://www.marinespecies.org/brachiopoda. Accessed 20 April 2014.

Emig C. Phylum Phoronida Hatschek, 1888. In: Zhang Z-Q, editor. Animal biodiversity: an outline of higher-level classification and survey of taxonomic richness. Zootaxa. 2011; 3148: 230.

Emschermann P. Loxokalypus socialis gen. et sp. nov. (Kamptozoa, Loxokalypodidae fam. nov.), ein neuer Kamptozoentyp aus dem nördlichen Pazifischen Ozean. Ein vorschlag zur neufassung der Kamptozoensystematik. Mar Biol. 1972; 12: 237-254.

Emschermann P. New species, taxonomist's delight and faunist's plight. On the variation range in Loxosomella vivipara Nielsen, 1966 (Entoprocta: Loxosomatidae). Zool Med Leiden. 2011; 85 (4): 69-77.

Eschmeyer WN, Fong JD. Pisces. In: Zhang Z-Q, editor. Animal biodiversity: an outline of higher-level classification and survey of taxonomic richness. Zootaxa. 2011; 3148: 26-38.

Garrity GM, Lilburn TG, Cole JR, Harrison SH, Euzéby J, Tindall BJ. Taxonomic outline of the Bacteria and Archaea, Release 7.7; 2007. Michigan State University Board of Trustees. doi: 10.1601/TOBA7.7.

Gill F, Donsker D, editors. IOC World Bird List (v 4.4); 2014. Available: http://www.worldbirdnames.org. Accessed 17 November 2014.

Giribet G, Kury AB. Phylogeny and biogeography. In: Pinto-da-Rocha R, Machado G, Giribet G, editors. Harvestmen: the biology of Opiliones. Cambridge, MA: Harvard University Press; 2007.

Goffinet B, Buck W, Shaw AJ. Morphology, anatomy, and classification of the Bryophyta. In: Goffinet B, Shaw AJ. Bryophyte biology, second edition. Cambridge, UK: Cambridge University Press; 2008.

Goto R, Okamoto T, Ishikawa H, Hamamura Y, Kato M. Molecular phylogeny of echiuran worms (Phylum: Annelida) reveals evolutionary pattern of feeding mode and sexual dimorphism. PLoS ONE. 2013; 8(2): e56809. doi:10.1371/journal.pone.0056809.

Guidetti R, Bertolani R. Phylum Tardigrada Doyère, 1840. In: Zhang Z-Q, editor. Animal biodiversity: an outline of higher-level classification and survey of taxonomic richness. Zootaxa. 2011; 3148: 96-97.

Guiry, MD, Guiry GM. AlgaeBase; 2014. World-wide electronic publication, National University of Ireland, Galway. Available: http://www.algaebase.org. Accessed 23 April 2014.

Guy L, Ettema TJG . The archaeal ‘TACK’ superphylum and the origin of eukaryotes. Trends in Microbiology. 2011; 19 (12): 580-587.

Harvey M. Smaller arachnid orders catalogue (SAOCat) database. In: Bisby FA, Roskov YR, Orrell TM, Nicolson D, Paglinawan LE, Bailly N, Kirk PM, Bourgoin T, Baillargeon G, Ouvrard D, editors. Species 2000 & ITIS Catalogue of Life: 2011 Annual Checklist, Digital resource; 2011. Available: http://www.catalogueoflife.org/annual-checklist/2011/.

Hausdorf B, Helmkampf M, Nesnidal MP, Bruchhaus I. Phylogenetic relationships within the lophophorate lineages (Ectoprocta, Brachiopoda and Phoronida). Mol Phylogenet Evolution. 2010; 55: 1121-1127.

Hodda M. Phylum Nematoda Cobb 1932. In: Zhang Z-Q, editor. Animal biodiversity: an outline of higher-level classification and survey of taxonomic richness. Zootaxa. 2011; 3148: 63-95.

Hooper JNA, Van Soest RVM, Pisera A. Phylum Porifera Grant, 1826. In: Zhang Z-Q, editor. Animal biodiversity: an outline of higher-level classification and survey of taxonomic richness. Zootaxa. 2011; 3148: 13-18.

ITIS—Integrated Taxonomic Information System; 2013. Available: http://www.itis.gov. Accessed 23 April 2013.

ITIS—Integrated Taxonomic Information System; 2014. Available: http://www.itis.gov. Accessed 21 April 2014.

James TY, Pelin A, Bonen L, Ahrendt S, Sain D, Corradi N, Stajich JE. Shared signatures of parasitism and phylogenomics unite Cryptomycota and Microsporidia. Curr Biol. 2013; 23: 1548-1553.

Jamieson B. On the phylogeny and higher classification of the Oligochaeta. Cladistics. 1988; 4: 367-401.

Janies D, Voight J, Daly M. Echinoderm phylogeny including Xyloplax, a progenetic asteroid. Syst. Biol. 2011; 60(4): 420-38. doi: 10.1093/sysbio/syr044. Epub 2011 Apr 27.

Janssens F, Christiansen KA. Class Collembola Lubbock, 1870. In: Zhang Z-Q, editor. Animal biodiversity: an outline of higher-level classification and survey of taxonomic richness. Zootaxa. 2011; 3148: 192-194.

Jiminez-Guri E, Philippe H, Okamura B, Holland PWH. *Buddenbrockia* is a cnidarian worm. Science. 2007; 317: 116-118.

Karpov SA, Mikhailov KV, Mirzaeva GS, Mirabdullaev IM, Mamkaeva KA, Titova NN, Aleoshin VV. Obligately phagotrophic aphelids turned out to branch with the earliest-diverging fungi. Protist. 2013; 164: 195-205.

Kenrick P, Crane PR. The origin and early evolution of plants on land. Nature. 1997; 389: 33-39.

Kirk P. (2013) Updated database version of Kirk P, Cannon P, Minter D, Stalpers J. Dictionary of Fungi, 10th Edition, CAB International, Oxon, UK; 2008.

Kristensen R. An introduction to Loricifera, Cycliophora, and Micrognathozoa. Integ and Comp Biol. 2002; 42: 641-651.

Kristensen R, van der Land J. Cycliophora; 2013. Available: World Register of Marine Species at http://www.marinespecies.org/aphia.php?p=taxdetails&id=22586. Accessed 9 July 2013.

Kroh A, Smith AB. The phylogeny and classification of post-palaeozoic echinoids. J Syst Palaeontol. 2010; 8:147-212.

Kroh A. Echinoidea; 2014. Available: World Register of Marine Species at http://www.marinespecies.org. Accessed 23 April 2014.

Lee J, Leedale G, Bradbury P, editors. An illustrated guide to the Protozoa, second edition: organisms traditionally referred to as Protozoa, or newly discovered groups. Volumes 1 & 2. Lawrence, KS: Society of Protozoologists; 2000.

Livezey BC, Zusi RL. Higher-order phylogeny of modern birds (Theropoda, Aves: Neornithes) based on comparative anatomy, II, analysis and discussion. Zool J Linn Soc. 2007; 149: 1-95.

Löffler FE, Yan J, Ritalahti KM, Adrian L, Edwards EA, Konstantinidis KT, Müller JA, Fullerton H, Zinder SH, Spormann AM. Dehalococcoides mccartyi gen. nov., sp. nov., obligate organohalide-respiring anaerobic bacteria relevant to halogen cycling and bioremediation, belong to a novel bacterial class, Dehalococcoidia classis nov., order Dehalococcoidales ord. nov. and family Dehalococcoidaceae fam. nov., within the phylum Chloroflexi. Int J Syst Evol Microbiol. 2013; 63: 625-635.

LPSN—List of prokaryotic names with standing in nomenclature; 2013. Available: http://www.bacterio.net. Accessed 7 July 2013.

Ludwig W, Euzéby J, Whitman WB. Road map of the phyla Bacteroidetes, Spirochaetes, Tenericutes (Mollicutes), Acidobacteria, Fibrobacteres, Fusobacteria, Dictyoglomi, Gemmatimonadetes, Lentisphaerae, Verrucomicrobia, Chlamydiae, and Planctomycetes; 2008. Available: http://bergeys.org/outlines/bergeys_vol_4_roadmap_outline.pdf.

Ludwig W, Euzéby J, Schumann P, Busse H-J, Trujillo ME, Kämpfer P, Whitman WB. Road map of the Actinobacteria; 2012. Available: http://bergeys.org/outlines/bergeys_vol_5_roadmap_outline.pdf.

Lynn D. The ciliate resource archive; 2002. Available: http://www.uoguelph.ca/~ciliates/classification/genera.html. Accessed 25 April 2013.

Lynn D. The ciliated Protozoa: characterization, classification, and guide to the literature, third edition. New York: Springer; 2008. 638 p.

Mah C. World Asteroidea Database; 2014. Available: World Register of Marine Species at http://www.marinespecies.org. Accessed 23 April 2014.

Maletz J. (2014) The classification of the Pterobranchia (Cephalodiscida and Graptolithina). Bulletin of Geosciences 89(3), ISSN 1214-1119.

Margulis L, Schwartz KV. Five kingdoms: an illustrated guide to the phyla of life on earth, third edition. New York: WH Freeman & Company; 1998.

Mayer G, Oliveira ID. Phylum Onychophora Grube, 1853. In: Zhang Z-Q, editor. Animal biodiversity: an outline of higher-level classification and survey of taxonomic richness. Zootaxa. 2011; 3148: 98.

Messing C. World list of Crinoidea; 2014. Available: World Register of Marine Species at http://www.marinespecies.org. Accessed 23 April 2014.

Mills C. Phylum Ctenophora: list of all valid species names; 2012. Available at: http://faculty.washington.edu/cemills/Ctenolist.html. Accessed 22 February 2014.

Minelli A. Class Chilopoda, Class Symphyla and Class Pauropoda. In: Zhang Z-Q, editor. Animal biodiversity: an outline of higher-level classification and survey of taxonomic richness. Zootaxa. 2011; 3148: 157-158.

Monks S, Richardson DJ. Phylum Acanthocephala Kohlreuther, 1771. In: Zhang Z-Q, editor. Animal biodiversity: an outline of higher-level classification and survey of taxonomic richness. Zootaxa. 2011; 3148: 234-237.

Moroz LL, Kocot KM, Citarella MR, Dosung S, Norekian TP, Povolotskaya IS, Grigorenko AP, Dailey C, Berezikov E, Buckley KM, Ptitsyn A, Reshetov D, Mukherjee K, Moroz TP, Bobkova Y, Yu F, Kapitonov VV, Jurka J, Bobkov YV, Swore JJ, Girardo DO, Fodor A, Gusev F, Sanford R, Bruders R, Kittler E, Mills CE, Rast JP, Derelle R, Solovyev VV, Kondrashov FA, Swalla BJ, Sweedler JV, Rogaev EI, Halanych KM, Kohn AB. The ctenophore genome and the evolutionary origins of neural systems. Nature. 2014. doi:10.1038/nature13400.

Narasingarao P, Podell P, Ugalde JA, Brochier-Armanet C, Emerson JB, Brocks JJ, Heidelberg KB, Banfield JF, Allen EE De novo metagenomic assembly reveals abundant novel major lineage of Archaea in hypersaline microbial communities. The ISME Journal. 2012; 6: 81–93.

National Center for Biotechnology Information: NCBI taxonomy browser; 2013. Available: http://www.ncbi.nlm.nih.gov/. Accessed 7 July 2013.

Nelson JS. Fishes of the world, fourth edition. Hoboken, NJ: John Wiley & Sons, Inc.; 2006.

Nielsen C. Entoprocta; 2013. Available: World Register of Marine Species at http://www.marinespecies.org/aphia.php?p=taxdetails&id=1271. Accessed 9 July 2013.

Norenburg J, Gibson R. World Nemertea Database; 2013. Available: World Register of Marine Species at http://www.marinespecies.org/aphia.php?p=taxdetails&id=152391. Accessed 9 July 2013.

Paulay G. Holothuroidea; 2014. Available: World Register of Marine Species at http://www.marinespecies.org. Accessed 23 April 2014.

Pawson DL. Phylum Echinodermata. In: Zhang Z-Q, Shear WA, editors. Linnaeus tercentenary: progress in invertebrate taxonomy. Zootaxa. 2007; 1668: 749-764.

Philippe H, Brinkmann H, Copley RR, Moroz LL, Nakano H, Poustka AJ, Wallberg A, Peterson KJ, Telford MJ. Acoelomorph flatworms are deuterostomes related to *Xenoturbella*. Nature. 2011; 470: 255-258.

Pirani J, Prado J. Embryopsida, a new name for the class of land plants. Taxon. 2012; 61: 1096-1098.

Poinar G. Fossil onychophorans from Dominican and Baltic amber: *Tertiapatus dominicanus* n. g., n. sp. (Tertiapatidae n. fam.) and *Succinipatopsis balticus* n. g., n. sp. (Succinipatopsidae n. fam.) with a proposed classification of the subphylum Onychophora. Invertebr Biol. 2000; 119: 104-109.

Qiu YL, Muramatsu M, Hanada S, Kamagata Y, Guo RB, Sekiguchi Y. Oligosphaera ethanolica gen. nov., sp. nov., an anaerobic, carbohydrate-fermenting bacterium isolated from methanogenic sludge, and description of Oligosphaeria classis nov. in the phylum Lentisphaerae. Int J Syst Evol Microbiol. 2013; 63: 533-539.

Read G, Fauchald K, editors. World Polychaeta database; 2014. Available: World Register of Marine Species at http://www.marinespecies.org/aphia.php?p=taxdetails&id=233983. Accessed 18 November 2014.

Renzaglia K, Villareal J, Duff RJ. New insights into morphology, anatomy, and systematics of hornworts. In: Goffinet B, Shaw AJ, editors. Bryophyte biology, second edition. Cambridge, UK: Cambridge University Press; 2008.

Reveal J. A checklist of familial and suprafamilial names for extant vascular plants. Phytotaxa. 2010 6: 1-402.

Reveal JL, Chase MW. APG III: bibliographical information and synonymy of Magnoliidae. Phytotaxa. 2011; 19: 71-134.

Riutort M, Alvarez-Presas M, Lazaro E, Sola E, Paps J. Evolutionary history of the Tricladida and the Platyhelminthes: an up-to-date phylogenetic and systematic account. Int J Dev Biol. 2012; 56: 5-17.

Roskov Y, Kunze T, Orrell T, Abucay L, Paglinawan L, Culham A, Bailly N, Kirk P, Bourgoin T, Baillargeon G, Decock W, De Wever A, Didziulis V, editors. Species 2000 & ITIS Catalogue of Life, 2014 Annual Checklist [DVD]; 2014. Species 2000: Naturalis, Leiden, the Netherlands.

Rouse G, Fauchald K. Cladistics and polychaetes. Zool Scr. 1997; 26:139-204.

Rousset V, Pleijel F, Rouse GW, Erseus C, Siddall ME. A molecular phylogeny of annelids. Cladistics. 2007; 23: 41-631.

Schuchert P. Holothuroidea; 2013. Available: World Register of Marine Species at http://www.marinespecies.org/aphia.php?p=taxdetails&id=123083. Accessed 10 July 2013.

Segers H. Phylum Rotifera Cuvier, 1817. In: Zhang Z-Q, editors. Animal biodiversity: an outline of higher-level classification and survey of taxonomic richness. Zootaxa. 2011; 3148: 231-233.

Shear W. Class Diplopoda de Blainville in Gervais, 1844. In: Zhang Z-Q, editor. Animal biodiversity: an outline of higher-level classification and survey of taxonomic richness. Zootaxa. 2011; 3148: 159-164.

Shimura J, Hiraki K, Garrity GM, editors. BIOS: Bacteriology Insight Orienting System (version Dec 2006). In: Bisby FA, Roskov YR, Orrell TM, Nicolson D, Paglinawan LE, Bailly N, Kirk PM, Bourgoin T, Baillargeon G, Ouvrard D, editors. Species 2000 & ITIS Catalogue of Life, 2011 Annual Checklist [DVD]; 2011. Species 2000: Reading, UK.

Sierra R, Matz M, Aglyamova G, Pillat L, Decelle J, Not F, de Vargas C, Pawlowski J. Deep relationships of Rhizaria revealed by phylogenomics: a farewell to Haeckel's Radiolaria. Mol Phylogenet Evol. 2012; 67: 53-59.

Silberfeld T, Rousseau F, De Reviers B. An updated classification of brown algae (Ochrophyta, Phaeophyceae). Cryptogam, Algol. 2014; 35 (2): 117-156.

Sørensen M. Phylum Kinorhyncha. In: Zhang Z-Q, editor. Animal biodiversity: an outline of higher-level classification and survey of taxonomic richness (Addenda 2013). Zootaxa; 2013; 3703: 63-66.

Sørensen MV, Giribet G. A modern approach to rotiferan phylogeny: combining morphological and molecular data. Mol Phylogenet Evol. 2006; 40: 585-608.

Steiner G, Kabat AR. Catalogue of supraspecific taxa of Scaphopoda (Mollusca). Zoosystema. 2001; 23(3): 433-460.

Stevens PF. Angiosperm phylogeny website, Version 12; 2014. Available: http://www.mobot.org/MOBOT/research/APweb/. Accessed 18 November 2014.

Stöhr S. Ophiuroidea. In: Stöhr S, O’Hara T, Thuy B, editors. World Ophiuroidea database; 2014. Available: World Register of Marine Species at http://www.marinespecies.org. Accessed 23 April 2014.

Stotler, R., Crandall-Stotler, B. Bryophytes: mosses, liverworts & hornworts; 2013. Available: http://bryophytes.plant.siu.edu/class.html. Accessed 10 July 2013.

Struck TH, Schult N, Kusen T, Hickman E, Bleidorn C, McHugh D, Halanych KM. Annelid phylogeny and the status of Sipuncula and Echiura. BMC Evol Biol. 2007; 7: 57.

Struck TH, Paul C, Hill N, Hartmann S, Hoesel C, Kube M, Lieb B, Meyer A, Tiedemann R, Purschke G, Bleidorn C. Phylogenomic analyses unravel annelid evolution. Nature. 2011; 471: 95-98.

Szeptycki A. Catalogue of the world Protura. Wydawnictwa Instytutu Systematyki I Ewolucji Zwierzat, Polskiej Akademii Nauk, Kraków; 2007.

Thollesson M, Norenburg JL. Ribbon worm relationships: a phylogeny of the phylum Nemertea. Proc R Soc Lond B Biol Sci. 2003; 270: 407-415.

Thuesen EV. Phylum Chaetognatha; 2009. Available: http://academic.evergreen.edu/t/thuesene/chaetognaths/chaettaxon.htm. Accessed 12 February 2014.

Thuesen EV. Chaetognatha; 2013. Available: World Register of Marine Species at http://www.marinespecies.org/aphia.php?p=taxdetails&id=2081. Accessed 12 February 2014.

Timm T. Oligochaeta; 2014. Available: World Register of Marine Species at http://www.marinespecies.org. Accessed 24 April 2014.

Timme RE, Bachvaroff TR, Delwiche CF. Broad phylogenomic sampling and the sister lineage of land plants. PLoS One. 2012; 7(1), e29696. doi:10.1371/journal.pone.0029696.

Tyler S. Polycladida; 2014. Available: World Register of Marine Species at http://www.marinespecies.org. Accessed 20 April 2014.

Tyler S, Schilling S. Phylum Xenacoelomorpha Philippe et al., 2011. In: Zhang Z-Q, editor. Animal biodiversity: an outline of higher-level classification and survey of taxonomic richness. Zootaxa. 2011; 3148: 24-25.

Tyler S, Schilling S, Hooge M, Bush LF, compilers. Turbellarian Taxonomic Database, Version 1.7; 2012. Available: http://turbellaria.umaine.edu.

Van Soest RWM, Boury-Esnault N, Hooper JNA, Rützler K, de Voogd NJ, Alvarez de Glasby B., Hajdu E, Pisera AB, Manconi R, Schoenberg C, Janussen D, Tabachnick KR, Klautau M, Picton B, Kelly M, Vacelet J, Dohrmann M, Cristina Díaz M. World Porifera database; 2013. Available: World Register of Marine Species at http://www.marinespecies.org/aphia.php?p=taxdetails&id=16481. Accessed 9 July 2014.

Wiley EO, Johnson GD. A teleost classification based on monophyletic groups. In: Nelson JS, Schultze H-P, Wilson MVH, editors. Origin and phylogenetic interrelationships of teleosts. München: Verlag Dr. Friedrich Pfeil; 2010.

Williams A, Carlson SJ, Howard C, Brunton C, Holmer LE, Popov L. A supra-ordinal classification of the Brachiopoda. Philos Trans R Soc Lond B Biol Sci. 1996; 351: 1171-1193.

Williams DM, Kociolek JP. Pursuit of a natural classification of diatoms: history, monophyly and the rejection of paraphyletic taxa. Eur J Phycol. 2007; 42: 313-319.

Wilson DE, Reeder DM. Class Mammalia Linnaeus, 1758. In: Zhang Z-Q, editor. Animal biodiversity: an outline of higher-level classification and survey of taxonomic richness. Zootaxa. 2011; 3148: 56-60.

Woese CR, Fox GE. Phylogenetic structure of the prokaryotic domain: the primary kingdoms. Proc Natl Acad Sci USA. 1977; 74 (11): 5088-5090.

WoRMS. Hemichordata; 2013. Available: World Register of Marine Species at http://www.marinespecies.org/aphia.php?p=taxdetails&id=1818. Accessed 10 August 2013.

WoRMS. Platyhelminthes; 2013. Available: World Register of Marine Species at http://www.marinespecies.org/aphia.php?p=taxdetails&id=793. Accessed 9 July 2013.

WoRMS. Annelida; 2013. Available: World Register of Marine Species at http://www.marinespecies.org/aphia.php?p=taxdetails&id=882. Accessed 10 August 2013.

Xiong Y, Gao Y, Yin W, Luan Y. Molecular phylogeny of Collembola inferred from ribosomal RNA genes. Mol Phylogenet Evol. 2008; 49: 728-735.

Yoon HS, Mueller KM, Sheath RG, Ott FD, Bhattacharya D. Defining the major lineages of red algae (Rhodophyta). J Phycol. 2006; 42: 482-492.

Zhang Z-Q, editor. Animal biodiversity: an outline of higher-level classification and survey of taxonomic richness. Zootaxa. 2011; 3148: 1-237.

Zhang Z-Q, editor. Animal biodiversity: an outline of higher-level classification and survey of taxonomic richness (Addenda 2013). Zootaxa. 2013; 3703: 1-82.
